# Bilateral Preseptal Cellulitis and Upper Eyelid Abscess Without Predisposing Factors: A Case Report and Pathogenic Hypothesis

**DOI:** 10.7759/cureus.76067

**Published:** 2024-12-20

**Authors:** Seyed Mohsen Rafizadeh, Mohammad Taher Rajabi, Amirhossein Aghajani, Amin Zand

**Affiliations:** 1 Department of Oculo-Facial Plastic and Reconstructive Surgery, Farabi Eye Hospital, Tehran University of Medical Sciences, Tehran, IRN

**Keywords:** bacteremia, cellulitis, hematogenous, periorbital, preseptal, sinusitis

## Abstract

Bilateral preseptal cellulitis without accompanying sinusitis or skin trauma is uncommon. In this report, we present a case of bilateral preseptal cellulitis and an upper eyelid abscess in an otherwise healthy child. A nine-year-old girl presented with severe and progressive bilateral swelling of the upper lids that showed an unsatisfactory response to medical treatments (intravenous ceftazidime and vancomycin) and warranted a referral to our facility. An orbital computed tomography scan and magnetic resonance imaging revealed bilateral preseptal soft tissue swelling with a collection but without intraorbital extension or paranasal sinus fullness. The most probable cause of the condition was considered to be transient bacteremia from an unknown source. Treatment involved administering broad-spectrum antibiotics and performing surgical drainage of the lid collections. Gram-positive cocci were identified in the smear of the drained components, although bacterial growth was not observed in the culture. Intravenous antibiotics were continued for five days, resulting in a remarkable decrease in swelling. In cases of atypical presentations of infectious preseptal cellulitis with abscess formation, particularly when there is bilateral involvement without common predisposing factors, the possibility of hematogenous spread of microorganisms should be carefully considered.

## Introduction

Preseptal cellulitis is an infection affecting the eyelid and surrounding tissue anterior to the orbital septum [1]. It is more commonly observed in children and typically manifests as a unilateral bacterial infection. Common predisposing factors for preseptal cellulitis include paranasal sinusitis and the contiguous spread of infection from the soft tissues of the face and ocular adnexa, particularly following trauma [2-4]. In some cases, this condition may be complicated by orbital subperiosteal abscess, orbital cellulitis, cavernous sinus thrombosis, or infectious meningitis, potentially leading to severe visual and life-threatening outcomes [5,6]. Generally, this condition resolves following conservative medical management with oral or intravenous antibiotics. However, in rare instances, it may be accompanied by an eyelid abscess or progress to orbital cellulitis, necessitating more aggressive interventions such as surgical drainage and debridement [7,8].

Here, we present a rare case of bilateral preseptal cellulitis characterized by abscess formation, occurring in a healthy child without concurrent sinusitis or skin trauma.

## Case presentation

The reporting of this study adheres to the CAse REport (CARE) guidelines [9]. All procedures were conducted in accordance with the principles outlined in the Declaration of Helsinki. Written informed consent for publication of the report and related images was obtained from the parents of the patient, and all patient details were de-identified. Ethical approval for case reports was not required by the institutional review board.

A nine-year-old female presented to the emergency department of Farabi Eye Hospital, Tehran, Iran with a history of fever, coryza, and headache, accompanied by severe bilateral upper lid swelling. The patient had no history of trauma or allergies. She initially presented with upper eyelid swelling approximately 10 days prior and was admitted to another center with a preliminary diagnosis of preseptal cellulitis. She was treated with intravenous antibiotics (ceftazidime and vancomycin) for 10 days. However, due to progressive eyelid swelling and an unsatisfactory response to treatment, she was referred to our tertiary center for further evaluation.

On examination, her vital signs were as follows: blood pressure 110/75 mmHg, axillary temperature 37.6 °C, respiratory rate 20 breaths per minute, and heart rate 105 beats per minute. There were no signs of sinusitis or periocular skin trauma. Additionally, no evidence of inflammatory or allergic skin conditions, such as dry skin, atopic dermatitis, or contact dermatitis, was observed. Furthermore, an oral examination revealed no dental abnormalities, including gingival abscesses.

**Figure 1:**
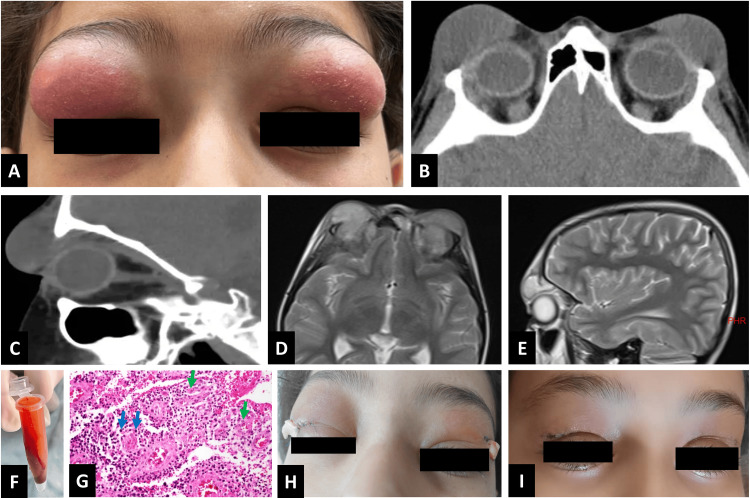
A: At baseline, severe bilateral swelling and erythema of upper lids. B, C: Non-contrast axial and sagittal computed tomography scans of the orbit showed bilateral abscess formation with soft tissue swelling of the lids. No evidence of paranasal sinus fullness was detected. D, E: Non-contrast axial and sagittal T2-weighted magnetic resonance imaging of the orbit revealed the same findings, without any other pathological disorders. F: Bloody and viscous serous discharge was drained from each upper eyelid, in the theater room. G: Histopathological evaluation revealed features of acute inflammation, indicated by polymorphonuclear cells (green arrows), alongside chronic inflammation, represented by lymphocytes (blue arrows), using hematoxylin and eosin staining at ×400 magnification. H: Day 4, decrease in swelling of both upper lids after medical treatment, surgical drainage, and Penrose drain tube insertion. I: Day 7, near complete recovery of the symptoms.

On ophthalmic examination, her best-corrected visual acuity was 20/20 in both eyes. Pupillary reactions were normal, and no relative afferent pupillary defect was detected. The patient exhibited bilateral upper lid swelling without restricted eye movements (Figure 1A). There were no remarkable findings of tenderness, necrosis, induration, or crepitation. Furthermore, no proptosis was present in each globe. The orbit was not noted to be tense or resistant to retropulsion. Slit-lamp examinations revealed no conjunctival chemosis or ciliary injection in either eye. A dilated fundus examination showed a clear vitreous with normal retina and optic discs.

Laboratory investigations from the previous admission revealed a white blood cell (WBC) count of 14.1 x 103/mcL with 53% neutrophils, an erythrocyte sedimentation rate (ESR) of 70 mm/h, and a qualitative C-reactive protein of 1+. Blood cultures were negative for microbial growth. During hospitalization at our center, a repeat WBC count showed 12.3 × 10³/ mcL with 62% neutrophils, an ESR of 45 mm/h, and a negative blood culture.

Orbital computed tomography (CT) scan without contrast revealed bilateral preseptal soft tissue swelling with abscess formation, without bone involvement or intraorbital extension. Furthermore, paranasal sinuses were clear (Figures 1B-1C). Subsequent orbital magnetic resonance imaging (MRI) without contrast confirmed preseptal soft tissue swelling and collection without any other abnormal findings (Figures 1D-1E).

Otorhinolaryngology consultation revealed mild erythema of the nasal, oral, and throat mucosa without exudative discharge or signs of necrosis. Therefore, the primary differential diagnosis was bilateral preseptal cellulitis with abscess formation.

The patient was admitted and treated with broad-spectrum antibiotics, including vancomycin (10 mg/kg/four times per day), ceftazidime (50 mg/kg/two times per day), and clindamycin (7.5 mg/kg/three times per day) following consultation with the infectious disease service. Using a tuberculin syringe with a 25-gauge needle, about 3 cc of mucopurulent material was aspirated from each eyelid. However, a day later, the collection was formed again. Therefore, surgical drainage was scheduled. In the theater room, approximately 4 cc of bloody serosanguinous discharge was drained from each upper eyelid and a Penrose drain tube was inserted and the incision site was sutured (Figure 1F). Pathological investigations of the discharge revealed gram-positive cocci, but bacterial culture after 48 hours did not yield any growth. Further pathological evaluations indicated signs of reactive acute-on-chronic inflammation (Figure 1G).

Intravenous antibiotics were continued along with a warm compress and massage. Oral prednisolone (15 mg/day) was added to the treatment regimen on day 3. By day 4, there was a significant improvement in lid swelling (Figure 1H), and the drain tube was removed as there was no discharge. On day 5, ophthalmic examinations and relevant laboratory tests, including complete blood count, were unremarkable, and the patient was discharged with oral clindamycin (150 mg/three times per day) and amoxicillin/clavulanic acid (375 mg/three times per day). A follow-up visit two days after discharge (day 7) revealed a remarkable decrease in swelling (Figure 1I). No recurrence of signs or symptoms of the disease was seen in the next follow-ups.

## Discussion

Conditions associated with eyelid swelling include superficial skin processes (such as atopic or contact dermatitis and skin neoplasms), inflammatory eyelid conditions (such as blepharitis, dacryoadenitis, dacryocystitis, or chalazion), local infections (such as hordeolum, preseptal cellulitis, or orbital cellulitis), and orbital mass effects (due to autoimmune or neoplastic lesions of the orbital cavity) [10]. In the presented case, based on the patient's history, clinical symptoms, and signs, as well as paraclinical evaluations, including a positive bacterial smear and response to broad-spectrum antibiotics, the most probable diagnosis was preseptal cellulitis.

Preseptal cellulitis typically presents as a unilateral condition and is more commonly observed in the pediatric population, although it can affect individuals of any age [11,12]. However, bilateral presentation is considerably rare [13-15]. Common predisposing factors for preseptal cellulitis include paranasal sinusitis and the contiguous spread of infection from the soft tissues of the face and ocular adnexa, particularly following trauma. According to a systematic review by Murphy et al., 68% of children with preseptal cellulitis had antecedent or current respiratory tract infections or sinusitis [16]. Similarly, another review found that among hospitalized children with preseptal cellulitis, the most common predisposing factors were upper respiratory infections (68%) and eyelid trauma (20%) [17]. In our case, although the patient exhibited symptoms of coryza, there were no obvious signs of respiratory infections, active sinusitis, or a history of periocular disease or trauma.

The most common bacterial pathogens of preseptal cellulitis are gram-positive cocci, particularly Staphylococcus and Streptococcus species [17,18]. Consistent with these findings, the smear of the drained material from the eyelid collections revealed gram-positive cocci. However, the culture was negative, which could be attributed to the prolonged (10-day) course of intravenous antibiotic treatment with ceftazidime and vancomycin administered at the previous center. Alternatively, a less common explanation for the negative culture could be laboratory errors [19]. Polymerase chain reaction (PCR) may have been a more sensitive method for detecting the causative microorganism in this case. However, due to the limited resources available in our laboratory, PCR was not performed.

Another, less common cause of preseptal cellulitis is melioidosis, a systemic infectious disease caused by the gram-negative bacterium Burkholderia pseudomallei. However, this diagnosis was not consistent with our case for several reasons: the specimen obtained from the eyelid abscess revealed gram-positive cocci, and melioidosis typically presents with more systemic symptoms and signs, such as sepsis, skin rashes, pneumonia, and lymphadenopathy, none of which were observed in our patient. Additionally, melioidosis is endemic in Southeast Asia and is not common in Iran, which generally has a warm and dry climate. Furthermore, our patient had no history of exposure to soil, water, or heavy rain, which are the most common predisposing factors for melioidosis [20].

There are hypotheses suggesting that the hematogenous spread of microorganisms from distant sources with no apparent predisposing factors may lead to preseptal cellulitis, with a tendency of bilateral presentation [14,21,22]. The hematogenous spread of microorganisms from distant sources to the periocular areas and orbit is well-recognized [22]. For instance, Argelich et al. reported a case of orbital cellulitis and endogenous endophthalmitis secondary to cholecystitis [23] while Muzzi et al. reported bilateral preseptal cellulitis following combined adenotonsillectomy and strabismus surgery in a healthy three-year-old boy [14]. In our case, the patient presented with fever and coryza symptoms without a history of sinusitis or periocular skin trauma. Thus, the hematogenous spread of microorganisms is the most reasonable cause of bilateral preseptal cellulitis with abscess formation in this instance, although the source of microorganisms spreading remained unidentified.

Preseptal cellulitis generally resolves with conservative medical management involving oral or intravenous antibiotics [17]. However, in rare cases, it can progress to orbital cellulitis with sight-threatening complications or even intracranial extension, leading to severe morbidities or even death [2,7]. Previous studies have indicated that the best predictors of post-septal disease in children with preseptal cellulitis are ophthalmoplegia, proptosis, and painful eye movements [16]. However, our patient did not exhibit any of these signs and did not progress to orbital cellulitis.

Surgical intervention is generally not required for patients with preseptal cellulitis unless there is an associated foreign body or eyelid abscess. Surgical drainage and debridement of a lid abscess can be performed through a small incision over the skin at a site of fluctuance, with consideration given to breaking loculations within the abscess cavity and inserting a drain tube to facilitate further drainage [7]. Similarly, in our case, due to an unsatisfactory response to intravenous antibiotics and the presence of preseptal abscess formation, incisions were made over the skin of both upper lids and drain tubes were inserted.

## Conclusions

In cases with atypical presentations of preseptal cellulitis, particularly those with bilateral involvement and lacking common predisposing factors, consideration of the hematogenous spread of microorganisms from distant sources is warranted and necessitates further evaluation. Furthermore, these cases may be at risk of abscess formation and require additional interventions, such as drainage, in addition to intravenous antibiotics.

## References

[1] Tritt A, Kay-Rivest E, Paradis T, Duval M (2019). Daily outpatient intravenous antibiotic therapy for the management of paediatric periorbital cellulitis, a retrospective case series. Clin Otolaryngol.

[2] Hauser A, Fogarasi S (2010). Periorbital and orbital cellulitis. Pediatr Rev.

[3] Santos JC, Pinto S, Ferreira S, Maia C, Alves S, da Silva V (2019). Pediatric preseptal and orbital cellulitis: a 10-year experience. Int J Pediatr Otorhinolaryngol.

[4] Sijuwola O, Adeyemo A, Adeosun A (2007). Orbital complications of rhinosinusitis. Ann Ib Postgrad Med.

[5] Bülbül L, Özkul Sağlam N, Kara Elitok G (2022). Preseptal and orbital cellulitis: analysis of clinical, laboratory and imaging findings of 123 pediatric cases from Turkey. Pediatr Infect Dis J.

[6] Asadigandomani H, Rajabi MT, Aghajani A (2024). The continuous rise in orbital subperiosteal abscess incidence in the Iranian pediatric population. Sci Rep.

[7] Lee S, Yen MT (2011). Management of preseptal and orbital cellulitis. Saudi J Ophthalmol.

[8] Afshar P, Aghajani A, Mohsenzadeh N, Heidari M, Rafizadeh SM, Abedinifar Z, Rajabi MT (2024). Pediatric orbital subperiosteal abscess outbreak in Iran: characteristics and causes. Graefes Arch Clin Exp Ophthalmol.

[9] Gagnier JJ, Kienle G, Altman DG, Moher D, Sox H, Riley D (2013). The CARE guidelines: consensus-based clinical case reporting guideline development. Headache.

[10] Sobel RK, Carter KD, Allen RC (2012). Periorbital edema. A puzzle no more?. Curr Opin Ophthalmol.

[11] Crosbie RA, Nairn J, Kubba H (2016). Management of paediatric periorbital cellulitis: our experience of 243 children managed according to a standardised protocol 2012-2015. Int J Pediatr Otorhinolaryngol.

[12] Upile NS, Munir N, Leong SC, Swift AC (2012). Who should manage acute periorbital cellulitis in children?. Int J Pediatr Otorhinolaryngol.

[13] Sheikh Y, Knipe H (2016). Bilateral preseptal periorbital cellulitis. Radiopaedia.org.

[14] Muzzi E, Parentin F, Pelos G (2013). Bilateral orbital preseptal cellulitis after combined adenotonsillectomy and strabismus surgery--case report and pathogenetic hypothesis. Int J Pediatr Otorhinolaryngol.

[15] Landeen KC, Mallory PW, Cervenka BP (2020). Bilateral ocular necrotizing fasciitis in an immunosuppressed patient on prescription eye drops. Cureus.

[16] Murphy DC, Meghji S, Alfiky M, Bath AP (2021). Paediatric periorbital cellulitis: a 10-year retrospective case series review. J Paediatr Child Health.

[17] Georgakopoulos CD, Eliopoulou MI, Stasinos S, Exarchou A, Pharmakakis N, Varvarigou A (2010). Periorbital and orbital cellulitis: a 10-year review of hospitalized children. Eur J Ophthalmol.

[18] Howe L, Jones NS (2004). Guidelines for the management of periorbital cellulitis/abscess. Clin Otolaryngol Allied Sci.

[19] Chao WC, Huang YW, Yu MC (2015). Outcome correlation of smear-positivity but culture-negativity during standard anti-tuberculosis treatment in Taiwan. BMC Infect Dis.

[20] Chang CY (2020). Periorbital cellulitis and eyelid abscess as ocular manifestations of melioidosis: a report of three cases in Sarawak, Malaysian Borneo. IDCases.

[21] Shapiro ED, Wald ER, Brozanski BA (1982). Periorbital cellulitis and paranasal sinusitis: a reappraisal. Pediatr Infect Dis.

[22] Durand ML (2022). Systemic bacterial infections and the eye. Albert and Jakobiec’s Principles and Practice of Ophthalmology. Springer International Publishing.

[23] Argelich R, Ibáñez-Flores N, Bardavio J (2009). Orbital cellulitis and endogenous endophthalmitis secondary to Proteus mirabilis cholecystitis. Diagn Microbiol Infect Dis.

